# Approach-Avoidance Decisions Under Threat: The Role of Autonomic Psychophysiological States

**DOI:** 10.3389/fnins.2021.621517

**Published:** 2021-03-31

**Authors:** James J. A. Livermore, Felix H. Klaassen, Bob Bramson, Anneloes M. Hulsman, Sjoerd W. Meijer, Leslie Held, Floris Klumpers, Lycia D. de Voogd, Karin Roelofs

**Affiliations:** ^1^Donders Institute for Brain Cognition and Behaviour, Radboud University, Nijmegen, Netherlands; ^2^Behavioural Science Institute, Radboud University, Nijmegen, Netherlands

**Keywords:** approach-avoidance, defensive freezing, threat processing, anxiety disorders, bodily states, autonomic nervous system, parasympathetic, cardiac deceleration

## Abstract

Acutely challenging or threatening situations frequently require approach-avoidance decisions. Acute threat triggers fast autonomic changes that prepare the body to freeze, fight or flee. However, such autonomic changes may also influence subsequent instrumental approach-avoidance decisions. Since defensive bodily states are often not considered in value-based decision-making models, it remains unclear how they influence the decision-making process. Here, we aim to bridge this gap by discussing the existing literature on the potential role of threat-induced bodily states on decision making and provide a new neurocomputational framework explaining how these effects can facilitate or bias approach-avoid decisions under threat. Theoretical accounts have stated that threat-induced parasympathetic activity is involved in information gathering and decision making. Parasympathetic dominance over sympathetic activity is particularly seen during threat-anticipatory freezing, an evolutionarily conserved response to threat demonstrated across species and characterized by immobility and bradycardia. Although this state of freezing has been linked to altered information processing and action preparation, a full theoretical treatment of the interactions with value-based decision making has not yet been achieved. Our neural framework, which we term the Threat State/Value Integration (TSI) Model, will illustrate how threat-induced bodily states may impact valuation of competing incentives at three stages of the decision-making process, namely at threat evaluation, integration of rewards and threats, and action initiation. Additionally, because altered parasympathetic activity and decision biases have been shown in anxious populations, we will end with discussing how biases in this system can lead to characteristic patterns of avoidance seen in anxiety-related disorders, motivating future pre-clinical and clinical research.

## Introduction

Effectively responding to a threatening situation poses a dilemma with ancient evolutionary origins: our survival may be at stake if we make a wrong decision. Do we approach the threat that may potentially harm us, or do we avoid it? This dilemma places vital selection pressures on our body, as we often need to execute a fast and appropriate response. While our body prepares to take the action, a characteristic pattern of heightened sympathetic arousal and parasympathetically driven immobility and bradycardia occurs (Nijsen et al., 1998; Bradley et al., 2001; Azevedo et al., 2005; Vila et al., 2007; Hagenaars et al., 2014; Gladwin et al., 2016): a bodily state referred to as threat-anticipatory freezing (Kozlowska et al., 2015; Roelofs, 2017). Freezing has been observed in both humans and non-human species, frequently in response to more distal threat (Blanchard et al., 2011; Mobbs et al., 2015). Human studies have shown that the magnitude of the freezing response is associated with altered information processing (Lojowska et al., 2015) and action preparation (Mobbs and Kim, 2015; Gladwin et al., 2016; Hashemi et al., 2019a,b; Rösler and Gamer, 2019). Given the importance of information gathering and action preparation in making adaptive responses in threatening situations, freezing could therefore be more than a passive state to avoid predator detection. In fact, freezing may facilitate value-based decision processes by optimizing threat perception and action preparation. Indeed, the magnitude of freezing has been shown to bias subsequent instrumental approach or avoid action (Ly et al., 2014). What remains unclear is *how* threat-anticipatory freezing affects these value-based decision-making processes.

Despite a potential role of threat-anticipatory freezing in value-based decision-making, current models of approach-avoidance decisions generally do not take bodily states of the decision maker into account. Mobilization of systems evolved for acute threat may underpin a variety of decision events in everyday life, where for example socially mediated sources of threat evoked by social hierarchies are present (Price, 2003). Therefore, not taking bodily states into account when trying to understand value-based decision-making potentially limits ecological validity. Moreover, clinical research shows that patients with anxiety-related disorders display chronically elevated autonomic activity (Brawman-Mintzer and Lydiard, 1997; Brosschot et al., 2016). Heightened autonomic activity likely underlies characteristic decision-making biases, including increased avoidance—a main symptom observed in anxiety patients (Hartley and Phelps, 2012). The high prevalence of anxiety-related disorders in the population (Bandelow and Michaelis, 2015), and the fact that individual differences in avoidance behaviors associate with variation in anxiety (Hulsman et al., 2021) highlights the importance of including bodily states (e.g., threat-anticipatory freezing) in value-based decision models.

In this paper we aim to outline the evidence for the role of threat-anticipatory freezing in value-based decision-making and instrumental action. Further, we will propose a new theoretical framework that incorporates a parasympathetically dominated threat-anticipatory freezing response into a decision-making model. Before presenting the new model, we provide an overview of the freezing state in the brain and body in the section “The Threat-Anticipatory Freezing State,” and describe evidence that threat-induced autonomic states exert influence on approach-avoidance decisions in the section “Threat-Anticipatory Freezing Is Associated With Information Gathering and Action Preparation.” We then more closely examine the case of value-based decisions in the section “Threat-Anticipatory Freezing Could Bias Value-Based Decisions,” and the separable processes of valuation and action preparation in the section “Threat-Anticipatory Freezing Could Bias the Switch to Action,” demonstrating how the freezing state may affect each of these in turn. Finally, in the section “The Threat State/Value Integration Model: A New Theoretical Neural Framework of Anticipatory Freezing on Approach-Avoidance Decisions Under Threat”, we outline our model, discuss how this model builds on existing theories of autonomic influence on decision processes, and outline a research agenda to further probe its predictions.

### The Threat-Anticipatory Freezing State

When faced with an acute threat, the body starts to prepare for action. The amygdala, especially the basolateral amygdala (BLA), is strongly connected to sensory input regions (Pitkänen et al., 1997; LeDoux and Daw, 2018) and thereby plays a key role in the initial *detection* and *processing* of the threat. Through intra-amygdala connections between BLA and the central nucleus (CeA), and projections from the CeA to the periaqueductal gray (PAG), hypothalamus, and rostral ventrolateral medulla, phasic autonomic activation is initiated. The hypothalamus and ventral medulla are involved in activating eccrine sweat glands and pupil dilation, heart and skeletal muscles (Jänig and McLachlan, 1992; Saha, 2005; Dawson et al., 2011; McDougal and Gamlin, 2014) serving the purpose of *initiating fast fight-or-flight reactions*. Alternatively, when threat is still at a distance and multiple action options are available, an increase in phasic parasympathetic activation typically occurs in concert with the sympathetic activation. During the resulting state of freezing, sympathetically driven heart rate increases are counteracted by projections from the ventrolateral PAG (vlPAG) through the vagus nerve to the heart, resulting in net bradycardia (Morgan and Carrive, 2001; Koba et al., 2016; Schipper et al., 2019). The vlPAG also suppresses phasic motor outputs through inhibition of motor neurons via the medulla, resulting in immobility but increased muscle tone from heightened arousal (Walker and Carrive, 2003; Kozlowska et al., 2015; Tovote et al., 2016). The increase in parasympathetic activation serves to put a brake on the already activated motor system. This leads, in addition to bradycardia, to physical immobility (Nijsen et al., 1998; Bradley et al., 2001, 2005, 2008; Roelofs et al., 2010; Hermans et al., 2013; Hagenaars et al., 2014; Löw et al., 2015; Gladwin et al., 2016; Bublatzky et al., 2017). It is this degree of motor reduction and bradycardia that mark out the parasympathetically dominated freezing state from sympathetically dominated fight-or-flight states (Kozlowska et al., 2015; Roelofs, 2017). When the switch from threat-anticipatory freezing to an action is made, parasympathetic withdrawal shifts the net balance of autonomic activity to sympathetic dominance, marked by tachycardia (Paton et al., 2005; Vila et al., 2007; Roelofs, 2017; Hashemi et al., 2019a). Neurally, subdivisions of the anterior cingulate cortex may play a role in switching autonomic modes, in particular the perigenual ACC (pgACC). The pgACC (and potentially the neighboring subgenual ACC) has a key role in controlling both branches of the autonomic nervous system, supported by extensive connections with hypothalamus and autonomic brainstem nuclei (Devinsky et al., 1995; Matthews et al., 2004; Critchley et al., 2005; Benarroch, 2012).

The freezing response may thus play a role particularly in circumstances where instrumental approach/avoidance actions may be possible, and where taking such actions may improve outcomes (accounting for the costs and benefits of action consequences) compared to automatic defensive reactions. Some examples are when more distal threat allows more time to calculate and prepare the next action (Mobbs et al., 2007; Brandão et al., 2008; Kozlowska et al., 2015; Roelofs, 2017; Wendt et al., 2017; Hébert et al., 2019), where levels of predator threat are intermediate (Eilam, 2005) and when there are no immediate escape routes available (Blanchard et al., 2011). Subsequent instrumental actions can be in line with the prepotent defensive reaction (i.e., engaging or escaping for fight and flight, respectively, or withholding action after freezing), but can also override automatic tendencies. Therefore, before an approach or avoid action is taken, the threat-anticipatory freezing state could provide a window in which value-based decision-making processes could occur.

## Threat-Anticipatory Freezing Is Associated With Information Gathering and Action Preparation

One theoretical account of a role for threat-anticipatory freezing in value-based decision-making, is that the parasympathetic brake on the sympathetically activated motor system allows further information gathering that can facilitate making the appropriate response (Friedman, 2007; Kozlowska et al., 2015; Roelofs, 2017). Thereby, threat-anticipatory freezing may allow for risk assessment (Blanchard et al., 2011) or resolve ambiguity and uncertainty (Eilam, 2005). Evidence for the involvement of freezing in information gathering comes from studies in humans showing that the magnitude of the freezing-related bradycardia is associated with changes in perception. For example, stronger freezing responses have been associated with preferential processing of low over high spatial frequency features of a visual stimulus (Lojowska et al., 2015, 2018) and reduced visual exploration of non-threat-relevant stimulus features (Rösler and Gamer, 2019).

Another example of how freezing may influence value-based decisions is that threat-anticipatory freezing can influence instrumental actions. Animal models have shown that threat-induced freezing can hamper active avoidance strategies (Martinez et al., 2013; Moscarello and LeDoux, 2013; Pavlova et al., 2020). Indeed, the transition from an automatic defense freezing reaction to successful instrumental avoidance requires a switch to action (Lázaro-Muñoz et al., 2010; Moscarello and LeDoux, 2013). Not all animals learn active avoidance strategies, and those that don’t may show persistent freezing in response to a Pavlovian threat cue (Choi and Kim, 2010; Lázaro-Muñoz et al., 2010). Lesioning the CeA, a region critically implicated in the freezing response increased active avoidance in those animals (Choi and Kim, 2010; Lázaro-Muñoz et al., 2010). In contrast, human studies show that freezing facilitates rapid responding (Jennings and van der Molen, 2005; del Paso et al., 2015; Gladwin et al., 2016; Hashemi et al., 2019a,b; Ribeiro and Castelo-Branco, 2019; Rösler and Gamer, 2019) and one study showed that threat-anticipatory freezing responses biased subsequent instrumental actions toward faster threat avoidance (Ly et al., 2014). Two recent studies (Hashemi et al., 2019a,b) manipulated threat of shock by using a task in which an avatar would shoot if an incorrect or delayed response was made. Under threat of shock, heightened bradycardia was observed, which was associated with an increase in immobility measured using a stabilometric force platform. Critically, the stronger the threat-anticipatory freezing response was, the faster participants responded in subsequent correct responses (Hashemi et al., 2019a). In the area of perceptual decision making, there is also evidence associating anticipatory bradycardia with faster decision making (Jennings and van der Molen, 2005; del Paso et al., 2015; Ribeiro and Castelo-Branco, 2019). Moreover, threat-anticipatory freezing responses are stronger when active responses are available to mitigate the threat, as compared to when it is not possible to escape the threat (Gladwin et al., 2016; Rösler and Gamer, 2019). To understand the apparent discrepancy between non-human animal and human findings, it is important to note that animal studies index freezing typically as the *duration* of immobility, while human studies typically index freezing as the *magnitude* of the freeze response (baseline-to-trough in terms of heart rate and/or immobility). Therefore, it remains unclear whether active avoidance in the above mentioned animal studies by Martinez et al. (2013) and Moscarello and LeDoux (2013) is in fact preceded by a transient state of freezing, and whether stronger magnitude of such initial freezing reaction may be related to faster subsequent responses as observed in human studies (e.g., Hashemi et al., 2019a). Further, the overall level of threat may be considerably higher in non-human animal studies than in humans, from ethically permissible shock magnitudes, possible selection bias in which participants sign up, and the ability of human participants to withdraw from studies. This raises the possibility that the action facilitation by freezing is only present in anticipation of intermediate threat levels.

**FIGURE 1:**
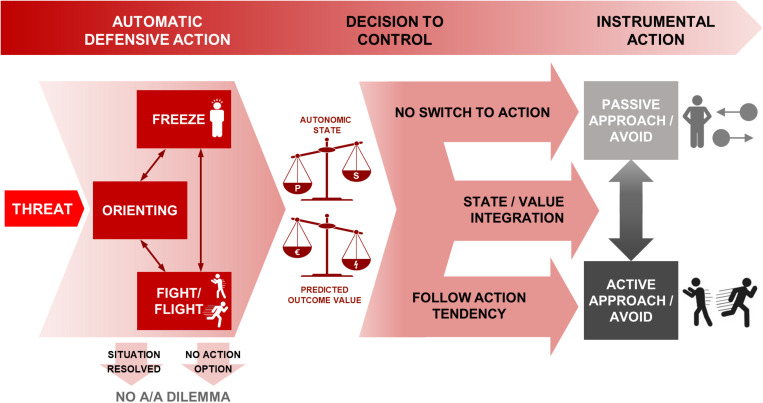
Schematic of processes from threat appearance to instrumental action decisions under approach-avoidance conflict. The appearance of threat gives rise to automatic defensive reactions in the first instance, including orienting, freezing and fight/flight. The situation may resolve itself at this stage, or no instrumental actions may be available, shown in the arrows terminating in no A/A dilemma. Otherwise, freezing and fight/flight are associated with prepotent action tendencies. Of those, freezing could be continued into inaction (top arrow, resulting in passive approach or avoidance) and fight/flight into approach/avoidance behavior (bottom arrow) without the need to override the prepotent action tendency. However, a decision can be made to control the prepotent tendencies in favor of an anticipated reward-punishment outcome. Both the decision to control, and the subsequent behavior if control is exerted, are based on the two scales shown: the predicted outcome from the assessed reward-punishment balance, and the autonomic balance of parasympathetic and sympathetic states (where for example more freezing requires a larger shift in order to take active action—see section “Threat-Anticipatory Freezing Could Bias the Switch to Action” for further details). These are integrated through state/value integration (middle arrow) to determine the choice of action—passive or active behavioral mode, and approach or avoidance action. P, parasympathetic; S, sympathetic; A/A, Approach/Avoidance.

Information gathering and action preparation are crucial aspects of making adaptive responses in threatening situations. Therefore, in the case when there is not an immediate defensive response (i.e., fight/flight), freezing allows enhanced information gathering and action preparation and potentially bias or facilitate the instrumental approach or avoid action. Figure 1 provides an overview of the time course of processes from threat appearance to instrumental action decisions.

## Threat-Anticipatory Freezing Could Bias Value-Based Decisions

One possible way threat-anticipatory freezing can influence the decision to approach or avoid, is by biasing the assessed value of the outcome. In a situation with potential danger, the organism needs to compare costs (i.e., the level of threat and the foregone reward) with benefits (i.e., potential reward and avoided threat), to select the appropriate instrumental approach. For example, escaping from potential predators incurs a cost in energy expenditure and foregone consumption opportunities which may itself prove survival-critical, while competition with social conspecifics for mating opportunities carries both the benefit of reproductive fitness and the danger of harm from physical conflict (Choi and Kim, 2010; Mobbs and Kim, 2015; Fendt et al., 2020).

Evidence for the notion that threat-anticipatory freezing may directly influence value computations of the approach-avoidance decision comes from studies in humans showing a relationship between bradycardia and decision-making. For example, in a non-threatening gambling task, bradycardia (as well as sympathetic driven skin conductance response; SCR) was shown to be higher in anticipation of disadvantageous relative to advantageous options for individuals that performed well, but not those who performed badly (Crone et al., 2004). Moreover, in an instrumental approach-avoidance study in humans, Ly et al. (2014) measured freezing responses by assessing reductions in body-sway to visually displayed angry (vs. happy) face primes, while participants were standing on a stabilometric force platform. This independent face prime was shown prior to an instrumental (monetary punished or rewarded) approach-avoidance decision. A critical observation in this study was that the magnitude of the threat-anticipatory freezing responses to angry faces biased subsequent behavior toward threat avoidance and against threat approach. As freezing responses to angry faces have been consistently linked to bradycardia (Roelofs et al., 2010; Niermann et al., 2017), this finding may be associated with the relationship between parasympathetic dominance during freezing and increased threat appraisal of angry faces (Bradley et al., 2001). These studies, however, have not directly investigated the link between threat-anticipatory freezing and value-based computation.

To date, relatively little research has investigated the influence of threat-anticipatory freezing and parasympathetic dominance on value-based decisions in threatening contexts, although recent work has specifically tested this link (Klaassen et al., 2021—discussed in the section “The Threat State/Value Integration Model: A New Theoretical Neural Framework of Anticipatory Freezing on Approach-Avoidance Decisions Under Threat”). There is some evidence, however, for an association between threat-induced *sympathetic* activation and subsequent decision-making. For example, using threat-of-shock tasks, associations were found between sympathetically driven pupil dilation and the processing of environmental uncertainty (Browning et al., 2015; de Berker et al., 2016), integrally related to subsequent learning and decisions. In addition, it was found that pupil dilation was associated with successful adaptation to changing contingencies (Browning et al., 2015). While these studies have provided important insight into autonomic contributions to decisions, without a concurrent parasympathetic measure it is not possible to determine the specificity of these effects to the sympathetic nervous system, particularly as freezing presents with phasic activity of both branches.

Changes in the balance between the sympathetic and parasympathetic nervous system during threat-anticipatory freezing may impact the reward-threat balance, by placing more weight on the aversive outcome. Indeed, it has been shown that threat and aversive value assessment occur largely in amygdala-PAG pathways (Seymour et al., 2005; Boll et al., 2013; McHugh et al., 2014; Roy et al., 2014), which is distinct from the pathway involved in appetitive value assessment, which occurs in striatal regions and the ventromedial prefrontal cortex (vmPFC; O’Doherty, 2004). Importantly, the amygdala-PAG pathway is also critically implicated in initiating threat-anticipatory freezing and thus accounts for a pathway that may increase the weight of the aversive outcome.

The impact of freezing on the reward-threat balance may also occur at the level of the integration. Indeed, threat assessment must be weighed against potential reward assessment that occurs in striatal regions and the vmPFC (Simon et al., 2010; Spielberg et al., 2013; Klumpers and Kroes, 2019). A region that may play a critical role in integrating value across modalities is the anterior cingulate cortex (ACC; Botvinick et al., 2004; Aupperle et al., 2015; Schlund et al., 2016). Importantly, the dorsal ACC (dACC) shows specific modulation of connectivity with the amygdala by exposure to threat (Carlson et al., 2013) and with the PAG by nociceptive stimuli (Hohenschurz-Schmidt et al., 2020). Although much literature localizes value integration in the dACC, some research also points to the neighboring subgenual area (Talmi et al., 2009; Park et al., 2011) and broader regions of the cingulate cortex (Roy et al., 2014; Gold et al., 2015).

This means that pathways involved in value integration overlap with pathways involved in threat detection and may thereby play a role in integrating the current bodily state (i.e., sympathetic vs. parasympathetic activation) and the value of the outcome (i.e., threat vs. reward).

## Threat-Anticipatory Freezing Could Bias the Switch to Action

Another way threat-anticipatory freezing can influence the decision to approach or avoid lays more at the level of the action. Namely, the weight of the threat-reward outcome must also be weighed against the cost to switch from parasympathetic to sympathetic activation.

For example, it was shown that in a non-threatening perceptual decision-making task, participants’ perceptual decisions on ambiguous stimuli were biased by the manipulated motor cost of response, despite no awareness that the motor cost was being incrementally altered (Hagura et al., 2017). This demonstrates that response initiation integrally involves the effort cost of behavior rather than being simply an output of higher-level decisions. The notion that the execution of an action may come at a cost may be relevant to understand dissociations of avoidance behavior across anxiety-related disorders. Namely, active avoidance (e.g., leaving a party to not engage in social interaction) may be more costly than passive avoidance strategies (e.g., not initiate eye contact to avoid a conversation). While depression and generalized anxiety are typically associated with passive avoidance, panic disorder and specific phobias are associated with active avoidance (Deakin and Graeff, 1991; Krypotos et al., 2015), so a closer understanding of this distinction both behaviorally and neurally may shed light on the distinctive features of these disorders.

Active and passive avoidance have also been associated with distinct neural pathways (Gozzi et al., 2010; Levita et al., 2012; Eldar et al., 2016; Tovote et al., 2016; Yu et al., 2016; Fadok et al., 2017). Animal models have identified neurons in the CeA responsible for switching behavioral responses to a threatening stimulus from freezing to overt approach-action (Gozzi et al., 2010; Moscarello and LeDoux, 2013; Fadok et al., 2017). A recent human study found connectivity between the pgACC, amygdala and PAG related to the switch from freeze to action (Hashemi et al., 2019a). This finding suggests that the ACC not only plays a role in conflict resolution but also in the switch from freezing-induced immobility to action. Intra-ACC connections to the perigenual region may then activate sympathetic responses to facilitate the chosen behavior, an idea supported by cytoarchitectural studies showing dense dorsal-perigenual ACC connections in monkeys supporting valenced responses to stimuli and initiation of active responses (Morecraft et al., 2012; Kim et al., 2018).

In contrast to the notion that freezing may enhance the cost of switching to action, in humans, stronger freezing has been observed in situations where an action has to be taken compared to when no action can be taken (Löw et al., 2015; Gladwin et al., 2016; Wendt et al., 2017) and the magnitude of freezing responses is associated with faster reaction time (Jennings and van der Molen, 2005; del Paso et al., 2015; Hashemi et al., 2019a,b; Ribeiro and Castelo-Branco, 2019).

Together these findings suggest that the switch from freeze to action also involves a value-based decision process and furthermore highlights the importance of incorporating the balance between parasympathetic and sympathetic activation into decision-making models.

## The Threat State/Value Integration Model: A New Theoretical Neural Framework of Anticipatory Freezing on Approach-Avoidance Decisions Under Threat

Taken together, the empirical work we reviewed in the previous sections allow us to formulate a new theoretical model of how threat-induced bodily states could affect value-base decision-making. The key proposal of this model is that the parasympathetically dominated state of freezing immediately following threat detection may be associated with biasing of subsequent decisions. The mechanism of this biasing may occur at three potential stages, corresponding to (1) the *processing of aversive valu*e, (2) *value integration*, and (3) *switching to action*. As the stages are along a common pathway, influences at each stage may be separable and interacting, and can be disentangled with computational and neural models.

**FIGURE 2:**
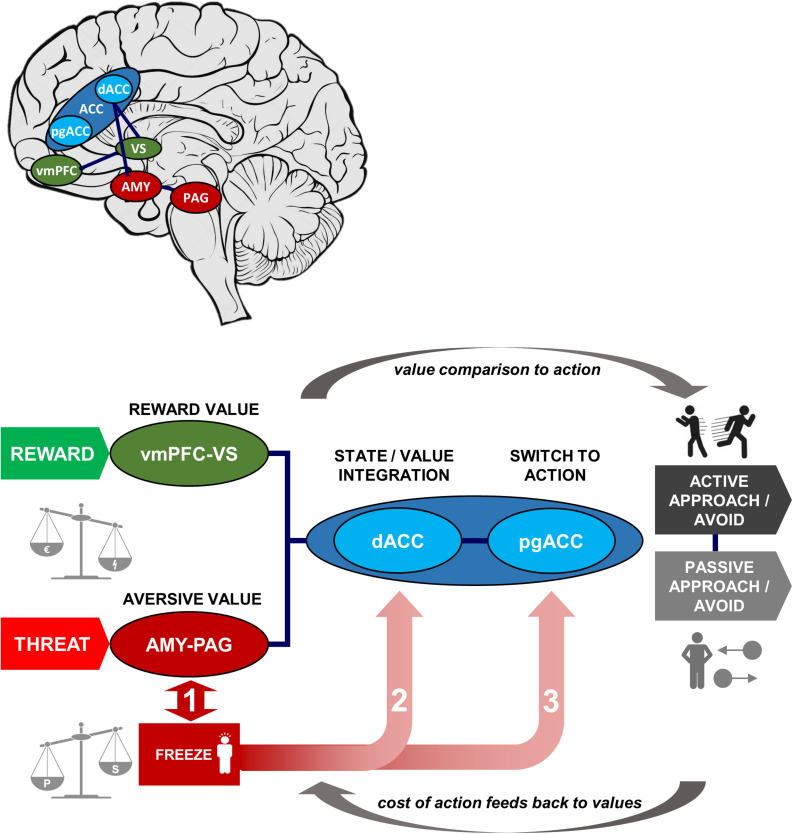
Schematic of Threat State/Value Integration (TSI) Model, with neural structures and functions involved in approach-avoidance action decisions under threat and illustrative map of locations within the brain. In our model approach-avoidance action decisions are determined not only by the predicted reward and aversive values of the action outcome but also by the costs of switching to action. Aversive and reward values are computed in amygdala-periaqueductal gray and ventromedial prefrontal cortex-ventral striatum subsystems, respectively (left of schematic), then compared in the anterior cingulate to output to action behavior (right of schematic; via sensorimotor regions, not shown). The degree of freezing is measured by the level of immobility and bradycardia, which are the result of the balance in sympathetic and parasympathetic activation. The degree of freezing may impact approach-avoidance action decisions at three possible stages (numbered in the figure): (1) altered aversive value assessment in threat-related pathways; (2) altered integration of values within the dorsal anterior cingulate; (3) altered cost of switching between parasympathetically dominated freezing and sympathetically dominated action in perigenual anterior cingulate cortex (pgACC). The circular arrows show the forward process of value comparison generating action, and the reverse process whereby action costs may retroactively affect value computations via a feedback loop. AMY, amygdala; PAG, periaqueductal gray; (d/pg)ACC, (dorsal/perigenual) anterior cingulate cortex; vmPFC, ventromedial prefrontal cortex; VS, ventral striatum. Green denotes reward and reward-related areas, red denotes threat and threat-related areas, and blue denotes areas of value integration and post-integration action switching.

Figure 2 illustrates these three decision stages through which freezing possibly affects the decision-making process (paths 1–3). Box 1 shows an exemplar model with corresponding effects on behavior that are predicted by influence at each stage.

**BOX 1 Exemplar model of freezing effects on approach-avoidance decisions at the three stages.**

The probability of an approach response is modelled here using a softmax function on values of reward and punishment, plus a dummy variable indicating whether the mode of response is active or passive in the current context. This produces three parameters that model the three stages of possible freezing influence on the approach-avoid decision. Estimation of individual subject parameters on behavioral data can then be used in parametric modelling of neural activity in the relevant brain areas discussed in Figure 2.

**(1)** At the stage of threat assessment, freezing may be associated with increased assessment of the aversive value of the current situation (higher value of 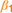), resulting in a lower likelihood of an approach action for a given reward-punishment balance. **(2)** At the value integration stage, freezing may modify the degree to which value assessment impacts behavior, resulting in more deterministic (higher 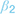), or stochastic (lower 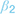) decisions at different levels of reward and punishment. **(3)** At the stage where assessment may lead to triggering or inhibition of action, biases in active and passive response modes may lead to differential behavior according to whether an active or passive response is required to approach or avoid the stimulus. An active (positive) response bias results in a greater probability of approach (positive 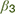), while a passive response bias produces the opposite effect (negative 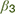).

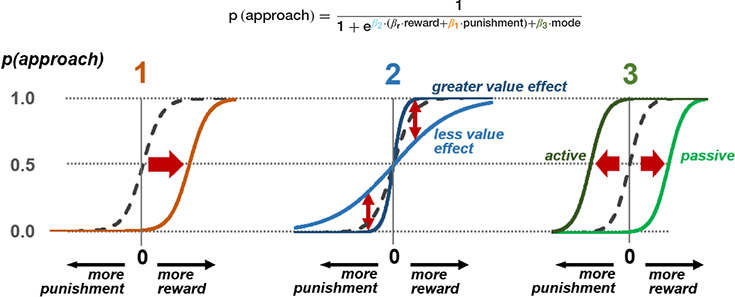

*Stage 1: computation of aversive value in the amygdala-PAG circuit.* This pathway is thought to be involved in carrying aversive information (e.g., pain) detected in the PAG to the amygdala (McHugh et al., 2014; Roy et al., 2014). Both the PAG and amygdala have additionally been implicated in initiation of defensive behavior, such as the freezing response (Hermans et al., 2013; Tovote et al., 2016; Hashemi et al., 2019a; Schipper et al., 2019). Thus, this circuit’s close relationship with initiation of threat-related behavior leads to a possible interaction between degree of freezing and predicted aversive value that may accordingly bias decisions and subsequent learning. Specifically, alterations in this circuit during freezing may be instantiated through inflated predictions of upcoming threat, amplifying the expected aversive value, correspondingly leading to an increase in avoidance behavior.

*Stage 2: integration of threat and reward values in dorsal ACC.* Comparison and integration of values across potential rewards and threat of aversive outcomes is thought to take place in the ACC, particularly the dorsal part. The dorsal ACC receives its value information from fronto-striatal regions and the amygdala, for appetitive and aversive outcomes respectively (Aupperle et al., 2015; Schlund et al., 2016), integrating these to determine the action to take. Through this route, freezing would not directly alter the value of any individual outcome (e.g., threat of shock), but rather bias the degree to which estimated reward and threat leads to behavior. This may therefore make behavior more deterministic (a steeper softmax function) if the effect of estimated reward-punishment balance is stronger, or more stochastic if it is weaker (a shallower function). Thus, taking together stages 1 and 2, freezing may affect evaluation of threats at lower and/or higher areas in the decision hierarchy.

*Stage 3: switch to action in perigenual ACC.* The third potential avenue of influence may lie in the switch from “passive” anticipation to action (approach/avoidance). Here, the pgACC is specifically implicated in switching from the parasympathetically dominated freezing state to a sympathetically dominant state for behavioral action (Hashemi et al., 2019a), in line with its role in autonomic control. Flexibility of this system, as well as the depth of the freezing state, may determine the relative cost of active behavior due to the effort of switching modes, and therefore bias value decisions depending on whether an action is required or not. This is in keeping with findings that decision making can be biased by the motor costs of responses (Hagura et al., 2017): response initiation is not simply an output of higher-level decisions but an interaction integrally involving the effort cost of behavior. This stage allows for freezing biases to be differentially evoked according to the type of response required for avoidance. Situations may require *active avoidance* (initiation of withdrawal behavior) or *passive avoidance* (inhibition of an approach response that results in non-engagement with the threatening stimulus), and these response modes are dissociable neurally (Gozzi et al., 2010; Levita et al., 2012; Eldar et al., 2016; Tovote et al., 2016; Yu et al., 2016; Fadok et al., 2017). Differential biases can be contrasted by using a task incorporating decisions of both types, and behavioral models incorporating both aversive value and switch costs. It is not always the case that freezing would bias to a higher action cost. In situations where freezing allows action preparation, it may in fact be associated with reduced action cost and bias toward active responding. Based on recent insights from both animal and human literature, individual differences in active and passive biases may impact the direction of effects in this stage (Moscarello and LeDoux, 2013; Klaassen et al., 2021).

In a recent study from our group (Klaassen et al., 2021) we developed the Passive-active Approach-avoidance Task (PAT) in which participants performed active and passive approach-avoid decisions. Heart rate, body sway, and skin conductance were measured throughout. In this task, participants were presented choices of varying monetary and shock levels, and required to make an approach-avoidance decision in both passive and active action conditions. Action contexts were created by manipulating the movement direction of the target to be approached/avoided. This study replicated previous findings showing a relationship between bradycardia and faster responding. It also demonstrated an association between freezing and the interaction between the response mode and subjective value of the choice options. This was found through computational modeling of these factors on the probabilities of approach and avoidance responses.

### The Threat State/Value Integration (TSI) Model’s Relationship With Existing Theoretical Frameworks

Theoretically, our theory fits with notions of two stage models (Mowrer, 1960), proposing that action invigoration is dissociable from value of the predicted outcome (Huys et al., 2011; Geurts et al., 2013; Guitart-Masip et al., 2014). Previous evidence is largely based on Pavlovian-instrumental transfer tasks, where the value of the Pavlovian response transfers to the instrumental action and the instrumental action itself does not occur under acute threat. Bach and colleagues have developed tasks where the approach-avoidance conflict involved potentially winning or losing points (Bach et al., 2014; Bach, 2015). However, unlike (for example) the threat of receiving an electrical shock, losing points is not a primary reinforcer. Threat of shock evokes activation at the level of the amygdala-PAG (Lojowska et al., 2018; Hashemi et al., 2019a; Schipper et al., 2019) where autonomic changes could influence instrumental approach-avoidance decisions. Our model is in line with several influential theories proposing that current bodily states can indeed impact approach-avoidance behavior (e.g., McNaughton and Corr, 2004; Porges, 2007; Strigo and Craig, 2016; Bach and Dayan, 2017). For example, Porges (2007) and Strigo and Craig (2016) both outline roles for the balance of sympathetic and parasympathetic influences in governing response to threat challenge, and in particular the regulatory role played by parasympathetic activity in maintaining healthy responses. McNaughton and Corr (2004) and Bach and Dayan (2017) focus on threat-specific systems, autonomic (sympathetic) arousal and their influences on approach-avoid behaviors. However, those theories do not explain at which stages threat-induced bodily states can impact approach-avoidance decisions. Nor do they make a distinction between sympathetic and parasympathetically dominated states. Lastly, it remains unknown what the neural implementation is of the effect of bodily states on decision-making. As such, our model extends previous accounts by including both branches of the autonomic nervous system.

### Predictions Based on the Model

Further testing of the predictions of the model will require research targeted specifically at the neural correlates of value-based approach-avoidance decisions under threat. Both neuroimaging work on humans, and translational studies with the greater specificity and causal testing allowed by animal methods such as optogenetics, can help to explore the interactions postulated by the model. We hypothesize that the interaction of freezing with predictions of aversive value in amygdala-PAG circuits and dACC will present with biases in active avoidance behavior through its alteration of the balance of assessed reward and aversive value. If interaction takes place on the process of switching to sympathetically driven action in pgACC, it will instead present with biases in passive avoidance, reflecting changes in the effort cost of switching to initiation of active behavior. Involvement of these brain circuits would be reflected in parametric changes in activity and connectivity in response to differing levels of threat. Further, we predict individual differences in biases related to these interactions, which may relate to clinical presentations on the more extreme ends. Previous research has indicated that clinical disorders may present with differential biases in passive and active response modes: panic disorder where a strong active avoidance bias is present, and generalized anxiety or ruminative presentations with a strong passive avoidance bias (Deakin and Graeff, 1991; Krypotos et al., 2015; White et al., 2016). Further research on clinical populations, or with animal models of these disorders, can determine whether these biases have explanatory power. Another important unresolved question regarding the model is the potential role of the bed nucleus of the stria terminalis (BNST). This region, sometimes referred to as the extended amygdala because of its close anatomical connections and overlapping function, plays a critical role in situations where threat is more ambiguous and/or distant in space or time (Lebow and Chen, 2016; Shackman and Fox, 2016; Klumpers et al., 2017). The BNST has strong connections to striatal and frontal regions involved in value calculations and motor control. Taken together, the BNST is anatomically very well placed to influence approach-avoidance decision making. So far however, its role in approach-avoidance decision making remains surprisingly unclear (Klumpers and Kroes, 2019). Further research would be needed to determine whether its role in approach-avoidance decisions fits within that of the amygdala as a whole, or whether these are distinguishable in levels of threat immediacy.

If connectivity between ACC and reward/threat-evaluative areas do support the comparison of these values under approach-avoidance decision making, this may allow for non-invasive brain stimulation to provide causal testing of this association. Recent work has shown that transcranial magnetic stimulation (TMS) can disrupt emotional (approach-avoidance) action control (Volman et al., 2011), while transcranial alternating current stimulation (tACS) can enhance this control through facilitation of coupling between prefrontal and sensorimotor areas (Bramson et al., 2020). Both interventions resulted in altered activity in fronto-amygdala-motor circuits. Behavioral interventions could focus on training a psychophysiological state compatible with bradycardia and increased heart rate variability prior to approach-avoidance decision making, such as recently developed in a biofeedback-integrated virtual reality game, where people make speeded approach avoidance decisions under acute threat (Brammer et al., 2021).

Whereas these more traditional brain stimulation techniques might not be ideal given our hypotheses on the involvement of deeper brain structures, new brain stimulation techniques that are capable of non-invasively manipulating brain activity in deep structures are currently being developed. For instance, it might be possible to change amygdala activity or modulate ACC functional connectivity with deeper brain structures using transcranial ultrasonic stimulation (macaques: Folloni et al., 2019; humans: Legon et al., 2018; Badran et al., 2020; Fini and Tyler, 2020), or by applying temporally interfering electrical fields (Grossman et al., 2017). These techniques can potentially be used to test causal predictions of the model by increasing or decreasing synchronization between structures. Another avenue for causal testing may lie in manipulation of neurochemical pathways related to the two branches of the autonomic nervous system. As these are generally differentiated between sympathetic (largely noradrenergic) and parasympathetic (cholinergic) nerve fibers (Sokolov et al., 1980; Paton et al., 2005; McDougal and Gamlin, 2014; Khan et al., 2016), the balance of their influences could be altered with drugs inhibiting or enhancing these pathways. In sum, this model provides the first neurocomputational account of the effect of the parasympathetically dominated threat-induced anticipatory freezing responses on decision making. We predict a set of behavioral and neural implications, which are now being tested. This model provides a fundamental framework of the interaction of physiological and neural systems across levels of the decision hierarchy in threatening contexts.

## Conclusion

We have reviewed substantial evidence of the relationship between threat-induced bodily states and decision making. While a considerable amount of work has shown that bodily states affect decision-making, a lack of integrative theoretical frameworks in this area hinders understanding of the exact routes by which sympathetic and parasympathetic balance changes influence decisions. We therefore provided a comprehensive neurocomputational account, the Threat State/Value Integration (TSI) Model, to integrate threat-induced bodily states with value-based decision-making models and generate concrete testable hypotheses. Better mechanistic understanding of how bodily states affect decision-making may ultimately inspire innovative training and therapy regimens, to optimize these decision processes in health and disease.

## Data Availability Statement

All datasets generated for this study are included in the article/supplementary material, further inquiries can be directed to the corresponding author/s.

## Author Contributions

JL and LV: writing, reviewing, and editing. FHK, BB, AH, SM, LH, and FK: reviewing and editing. KR: writing, reviewing, editing, and giving final approval. All authors contributed to the article and approved the submitted version.

## Conflict of Interest

The authors declare that the research was conducted in the absence of any commercial or financial relationships that could be construed as a potential conflict of interest.
